# Development of Biphasic Culture System for an Entomopathogenic Fungus *Beauveria bassiana* PfBb Strain and Its Virulence on a Defoliating Moth *Phauda flammans* (Walker)

**DOI:** 10.3390/jof11030202

**Published:** 2025-03-05

**Authors:** Yi-Ping Gao, De-Xiang Shi, Yuan-Hao Li, Xiong Zhao He, Xiao-Yun Wang, Kai Lin, Xia-Lin Zheng

**Affiliations:** 1 Guangxi Key Laboratory of Agro-Environment and Agric-Products Safety, College of Agriculture, Guangxi University, Nanning 530004, China; gaoyiping1212@163.com (Y.-P.G.); sdx803@foxmail.com (D.-X.S.); l18137818978@163.com (Y.-H.L.); wxy8771@163.com (X.-Y.W.); linkai@gxu.edu.cn (K.L.); 2 School of Agriculture and Environment, Massey University, Private Bag, Palmerston North 4410, New Zealand; x.z.he@massey.ac.nz

**Keywords:** *Beauveria bassiana*, fermentation technology, mycelium growth, sporulation, conidial validity and virulence, prolonging storage

## Abstract

*Beauveria bassiana* PfBb is a new strain with high host specificity to the target pest *Phauda flammans*. We conducted a series of experiments to optimize the biphasic fermentation system of *B. bassiana* PfBb by screening the medium compositions and fermentation environmental conditions in both liquid and solid fermentations. In the liquid fermentation, glucose and yeast extract with a C:N ratio of 17:1 were the optimal carbon and nitrogen sources, respectively, for *B. bassiana* PfBb mycelium growth and blastospore production, and liquid fermentation with an inoculation concentration of 1 × 10^8^/mL and an inoculum content of 50 mL conidial suspension, at 180 rpm/min rotation speed, pH 7 and 26 °C, favored mycelium growth. However, additional trace elements did not significantly improve liquid fermentation. In the solid fermentation, wheat bran and chaff at a ratio of 8:2 were identified as the best substrates that facilitated *B. bassiana* PfBb sporulation and conidial germination, and optimal substrates with 20% inoculum content, 50% water content, and 3-day fermentation in darkness had the highest conidia yield. The resulting conidia, stored at −20, 4, and 20 °C for one year, did not significantly change the water content, and with prolonged storage duration, conidial germination was significantly higher at −20 and 4 °C. Moreover, conidia stored at 4 °C for one year maintained its validity and virulence, which were toxic to all instar larvae of *P. flammans*. Our results provide essential support for the commercial production of *B. bassiana* PfBb-based biopesticides.

## 1. Introduction

Entomopathogenic fungi cause mortality of insect pests through infections and suppress pest populations in nature through epizootics. To date, more than 100 commercial biopesticides based on entomopathogenic fungi have been developed and widely used as sustainable biocontrol agents in integrated pest management (IPM) programs [1,2,3,4,5,6,7]. For instance, the entomopathogenic fungus *Beauveria bassiana* (Balsamo-Crivelli) Vuillemin (Hypocreales: Cordycipitacae) could attack a broad range of insect pests among the orders of Lepidoptera, Coleoptera, Hemiptera, Orthoptera, and Diptera [8], and is applied for pest management in greenhouses and fields [4,6,9]. However, it is highly challenging to optimize the fungal fermentation technology to produce adequate quantities of high-quality conidia and maintain the validity and virulence of stored conidia for a long storage period.

Biphasic fermentation can reduce processing time and increase production yields of entomopathogenic fungi on an industrial scale [10] and is thus commonly applied for mass production for biological control programs [11,12,13,14]. In biphasic fermentation, fungi are first grown in a liquid culture medium to produce metabolic active blastospores, which serve as inocula and are then inoculated on the solid culture substrates to produce aerial conidia [15,16]. Blastospores are a type of fungal spore produced asexually by budding, while conidia are asexual, non-motile spores produced by blastospores [7]. Blastospores can be mass-produced efficiently in liquid cultures, enabling cost-effective industrial-scale production [17,18]. Furthermore, blastospores germinate rapidly upon contact with insect hosts, accelerating infection compared to conidia [19]. However, blastospores exhibit poor tolerance to field conditions, particularly UV radiation and desiccation [19,20]. In contrast, solid-state fermentation enables cost-effective, scalable conidia production [21], and conidia exhibit superior tolerance to UV radiation, desiccation, and temperature fluctuations, ensuring persistence in field applications [22,23], and their robustness allows long-term storage and formulation into stable bioinsecticidal products [17,20]. However, production of conidia in solid-state fermentation is labor-intensive and less efficient than liquid culture methods [14], and the slower germination of conidia may potentially reduce immediate efficacy compared to blastospores [20]. The biphasic culture system shows distinct advantages, including precise control of nutritional and environmental factors, tailoring of the medium for optimal spore production, increasing of conidia production, recycling of inert substrate after removal of conidia and reprocessing, promotion of biomass generation, shortening of fermentation time, minimization of contamination risks, provision of a scalable and robust platform for mycopesticide production, etc. [7,11,14,15,24,25]. Large-scale production of entomopathogenic fungi as insect biocontrol agents using biphasic fermentation will meet the increasing demands of agricultural producers and commercial companies.

It is generally known that optimizing liquid or solid fermentation can offer more cost-effective scaling capabilities under controlled nutritional and environmental conditions [14,24,25,26,27,28,29,30,31,32,33,34]. However, to develop cost-effective large-scale production processes, it is essential to identify the medium materials and environmental factors that favor mycelial growth, sporulation, and conidia yield in either liquid or solid fermentation. The key parameters for optimizing industrial fermentation of pathogenic fungi may include nutrients, temperature, hydrogen ion concentration (pH), rotation speed, and inoculum size in liquid fermentation [30,34,35,36,37,38,39], and solid substrate composition, water content, aeration, inoculum size, temperature, lighting, and fermentation period in solid fermentation [14,31,32,34,40,41,42,43,44,45]. However, those key parameters for fungal fermentation are not identical but species- or even strain-specific [46,47,48].

In addition to designing production systems with a high yield of entomopathogenic fungi, the stability, validity, infectivity, and virulence of conidia in the resulting biopesticides are key parameters to determine the success of biocontrol programs, and thus must be considered during process development [49]. It is well known that the key quality parameters of conidia are affected by external environmental factors (e.g., temperature and humidity). Storage at low temperature and low humidity is essential to maintain the conidial quality [50,51,52], extend the shelf life, and help control the cost of biopesticide production [53]. Extending the shelf life of microbial pesticides will guarantee the potential large-scale applications in pest management [38,54,55,56].

*Phauda flammans* (Walker) (Lepidoptera: Phaudidae) is a serious pest of *Ficus* trees (Urticales: Moraceae) in China [57] and India [58]. Larvae of *P. flammans* feed on leaves and phloem at the tender tips of *Ficus* trees over large areas, eventually causing plant death [59]. Several potential biological control measures, such as nuclear polyhedrosis virus [60], parasitoids [61], and sex pheromones of *P. flammans* have been reported, but rarely applied, to control this pest [62]. Recently, we have identified a promising Chinese isolate of *B. bassiana* named PfBb that is highly effective in infecting and killing *P. flammans* larvae [63,64]. *B. bassiana* PfBb is even toxic to non-target species, for example, the important agricultural pest, fall armyworm, *Spodoptera frugiperda* (J. E. Smith) (Lepidoptera: Noctuidae), indicating its high potential in pest control on a large scale [65]. Our previous study confirms that this *B. bassiana* PfBb strain is more pathogenic to *P. flammans* larvae than other commercially available entomopathogenic fungi [63].

In this study, we carried out a series of experiments to optimize the nutrients and environmental conditions for *B. bassiana* PfBb growth and blastospore production in liquid culture fermentation, selected the optimum substrates determined the optimum medium environmental conditions for conidiation in solid culture fermentation, and finally, tested the conidia validity and its virulence on different instar larvae of *P. flammans* after a prolonging storage. Our results deliver knowledge to facilitate the mass production of *B. bassiana* PfBb.

## 2. Materials and Methods

### 2.1. Fungi

*Beauveria bassiana* PfBb used in this study was isolated from infected *P. flammans* larvae, collected in Hechi City, Guangxi, China [64]. The *Beauveria bassiana* PfBb strain was inoculated and cultured on potato dextrose agar, which was stored at −80 °C before using for experiments.

### 2.2. Insects

Mature *P. flammans* larvae (n = 2000) were collected on *Ficus benjamina* L. in Nanning City (108.21° E, 22.64° N), Guangxi Zhuang Autonomous Region, China, from June to July 2022. Fifteen *P. flammans* larvae were reared on fresh *F. benjamina* leaves in a transparent plastic container (20 cm diameter × 15 cm height) with 20–30 pinholes (1 mm diameter) for ventilation. The plant leaves were replaced with fresh ones daily until pupation. The pupae were kept individually in plastic tubes (2.7 cm diameter × 12 cm height) with 10–15 pinholes (1 mm diameter) on the lids. Newly emerged moths were paired and placed in a net cage (80 cm length × 80 cm width × 100 cm height) for mating. A potted *F. benjamina* seedling (60 cm height) was maintained in the cage as the oviposition substrate. The hatched larvae were reared as mentioned above until they developed into the desired instar stages used for the experiments. The insects were maintained at 25 ± 1 °C, 75 ± 5% relative humidity, and a photoperiod of 13L/11D (light/dark).

### 2.3. Optimization of Liquid Fermentation for B. bassiana PfBb

#### 2.3.1. Preparation of Standardized Blastospore and Conidial Suspension

Prior to the experiment, the *B. bassiana* PfBb strain was cultured on the potato dextrose agar (PDA) medium, composed of 200 g potato, 20 g glucose, 15 g agar, and 1000 mL water with a natural pH of 7, at 25 ± 1 °C, 80 ± 5% RH, and a 12L/12D photoperiod for 14 days. After this time, the conidia were harvested from the surface of the culture medium. The conidial concentration was measured using a hemocytometer and then adjusted to 1 × 10^7^ conidia/mL using 0.05% Tween-80 solution to obtain the standardized blastospores and conidial suspension for experiments. Conidium counting was performed under a microscope (CX21, Olympus Optics, Tokyo, Japan).

#### 2.3.2. Optimization of Nutrients in Liquid-State Culture Medium

In this experiment, a liquid-state culture medium, composed of 40 g glucose, 10 g peptone, and 1000 mL water with a pH of 7, was applied to optimize the carbon and nitrogen nutrients for *B. bassiana* PfBb growth. To optimize the carbon nutrient, we replaced glucose in the liquid-state culture medium with an equal amount (40 g) of fructose, sucrose, maltose, or mannitol as the carbon source, but maintained the 10 g peptone as the nitrogen source in the medium. To optimize the nitrogen nutrient, we replaced peptone in the liquid-state culture medium with an equal amount (10 g) of yeast extract, beef extract, corn flour, or sodium nitrate as the nitrogen source, but maintained the 40 g glucose as the carbon source in the medium. A quantityof 10 mL of standardized conidial suspension as prepared above were added into a 250 mL flask, maintaining 100 mL liquid-state culture medium containing one of the test carbon or nitrogen sources. The flask maintaining the test medium was placed on a rotary shaker (HNY-100B, Erno Instruments Co., Ltd., Tianjin, China) with a rotation speed of 150 rpm/min and incubated at 26 °C for 3 days, after which the incubated medium was shaken well. The blastospore concentration was quantified with a hemocytometer under the microscope. To determine the mycelium dry weight, the incubated liquid medium was filtered in a vacuum filter (SHZ-DIII, Ruide, Zhengzhou, China) through a filter paper placed on a Buchner funnel. The filtered samples on the filter paper were placed into a drying oven (GPX-9052, Yuejin Co., Ltd., Shanghai, China) set at 80 °C, dried until constant weight, and then weighed using an analytical balance (AR-224CN, Aarhus Instruments Co., Ltd., Shanghai, China). The liquid-state culture medium without standardized conidial suspension added served as control. There were three replicates for control and each test of carbon or nitrogen sources.

Based on the results of the above experiment, a further experiment was conducted to optimize the concentrations of the best carbon and nitrogen sources for the liquid medium. By using the same methods described above, the mycelium dry weight and blastospore concentration were evaluated at varying glucose concentrations of 20, 30, 40, 50, and 60 g/L with a constant yeast extract concentration of 10 g/L, and at varying yeast extract concentrations of 5, 10, 15, 20, and 25 g/L with a constant glucose concentration of 40 g/L. The carbon content in glucose is 40%, and carbon and total nitrogen contents in yeast extract are 40% and 10%, respectively [66]. Therefore, the carbon-to-nitrogen ratios (C:N) in the above tested glucose concentrations were 9:1, 13:1, 17:1, 21:1, and 25:1, respectively, and those in yeast extract concentrations were 33:1, 17:1, 11.7:1, 9:1, and 7.4:1, respectively. There were three replicates for each nutrient concentration test.

**Figure 1:**
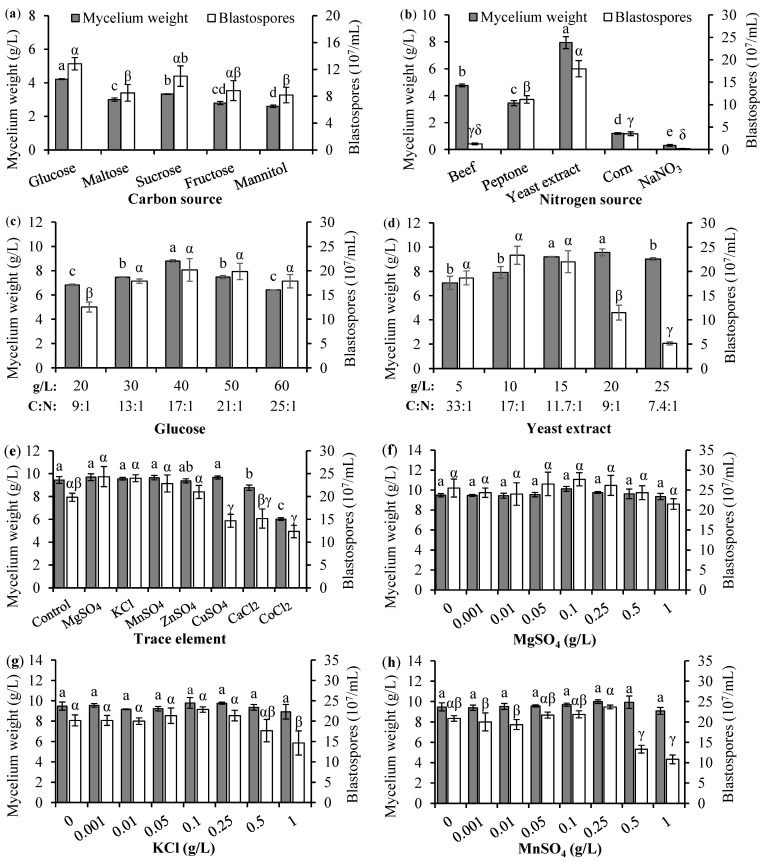
Mean (±SE) mycelium dry weight and blastospore number of *B. bassiana* PfBb growing in liquid culture medium affected by carbon source (**a**), nitrogen source (**b**), glucose concentration (**c**), yeast extract concentration (**d**), trace element (**e**), MgSO_4_ concentration (**f**), KCl concentration (**g**), and MnSO_4_ concentration (**h**). For mycelium weight, columns with the same English letters are not significantly different; and for blastospore number, columns with the same Greek letters are not significantly different (LSD test: *p* > 0.05).

To determine the type and concentration of trace elements suitable for liquid culture, the selected carbon and nitrogen sources and their concentrations (i.e., 40 g glucose/L and 15 g yeast extract/L) were used to prepare the liquid medium for this test. Each test medium was supplemented with 0.1 g of KCl, MgSO_4_, CaCl_2_, CuSO_4_, ZnSO_4_, MnSO_4_, or CoCl_2_. Three trace elements (i.e., MgSO_4_, KCl, and MnSO_4_; Figure 1) that potentially promoted *B. bassiana* PfBb growth were selected to test the effects of various concentrations (i.e., 0, 0.001, 0.01, 0.05, 0.1, 0.25, 0.5, and 1 g/L) on blastospore production. There were three replicates for each element and each concentration. The mycelium dry weight and blastospore concentration were measured as described above.

#### 2.3.3. Development of Environmental Conditions for *B. bassiana* PfBb Growth in Liquid Culture Medium

To improve the liquid culture medium, the effects of inoculation concentration (1 × 10^5^, 1 × 10^6^, 1 × 10^7^, 1 × 10^8^, and 1 × 10^9^ conidia/mL), inoculation volume of culture medium (50, 75, 100, 125, and 150 mL), agitation speed (120, 150, 180, 210, and 240 rpm/min), temperature (20, 23, 26, 29, and 32 °C) and pH (5, 6, 7, 8, and 9) on *B. bassiana* PfBb growth and reproduction were tested. In addition to the above test elements, the standard culture medium (i.e., 40 g glucose/L, 15 g yeast extract/L, 0.1 g KCl/L, 0.25 g MnSO_4_/L, 0.1 g MgSO_4_/L, and 1000 mL water with a pH of 7) was prepared; 10 mL standard conidial suspension (1 × 10^7^ conidia/mL) was added to a 250-mL flask maintaining 100 mL of standard culture medium, and the flask was then placed on a rotary shaker at 150 rpm/min and incubated at 26 °C for 3 days. The mycelium dry weight for each test condition was determined as described in Section 2.3.2. Each test was repeated three times.

### 2.4. Optimization of Solid Fermentation for B. bassiana PfBb

#### 2.4.1. Selection of Substrates for Solid Fermentation

To optimize the solid-state fermentation for conidia production of *B. bassiana* PfBb, we individually tested various types of substrates: (1) wheat bran, corn flour, beer draff, rice, and soybean meal that still retained a significant amount of nutritional components for fungal growth, and (2) bagasse, peanut shell, chaff, tobacco stem, and coir that might improve the physical structure of the culture medium. A test substrate (80 g) was mixed with 80 mL water and bagged in a sealed bag (40 cm length × 28 cm width) (BKMAM Biotechnology Co., Ltd., Changsha, China). For the rice substrate, 100 g rice was soaked in boiling water for 30 min and air dried before being mixed with water. The bagged medium was sterilized in an autoclave at 121 °C under 15 psi for 20 min, after which it was naturally cooled under laboratory conditions. The solid medium (80 g) was inoculated with 20 mL conidial suspension (1 × 10^8^ conidia/mL). The inoculated medium or substrate was incubated at 26 °C under dark conditions for the three days and then under light conditions for nine days. The incubated medium or substrate with *B. bassiana* PfBb conidia was air-dried at 30 °C for 24 h, then pulverized using a grinder (200T Boou Hardware Products Co., Ltd., Jinhua, China) and passed through an 80-mesh sieve to obtain conidia powder.

To determine the conidia yield, 1 g of BI (biological indicator) powder was dissolved with 100 mL of 0.05% Tween-80 solution, and the conidia number was counted using the hemocytometer under the microscope. To assess the conidial germination rate, 1 g BI powder was dissolved in 100 mL 0.05% Tween-80 solution, diluted to 1 × 10^7^ conidia/mL suspension, and then inoculated into liquid culture comprising 4% glucose and 1.5% yeast extract. The liquid culture was incubated at 26 °C for 24 h. Conidia germination rate was observed using a hemocytometer under the microscope. Conidia with their germinal tube reaching approximately half the diameter of the conidia were considered as germination [67]. Each treatment was replicated three times.

An additional experiment was conducted to determine the best culture medium-to-substrate ratio (i.e., wheat bran-to-chaff ratio), and the conidia yield was evaluated at various wheat bran-to-chaff ratios of 9:1, 8:2, 7:3, 6:4, and 10:0 (100 g in total). After mixing with 100 mL water, the bagged blend was sterilized in an autoclave as mentioned above. A quantity of mL conidial suspension (1 × 10^7^ conidia/mL) was inoculated onto the sterilized solid medium. The inoculated medium was then incubated at 26 °C under a 12L/12D photoperiod for 10 days, and the conidia yield was determined.

#### 2.4.2. Development of Conditions for *B. bassiana* PfBb Growth in Solid-State Medium

To improve the solid-state medium, the conidia yield was detected at the optimum ratio of conidial suspension to substrate inoculation content (proportion of fermented optimal-liquid medium to solid substrate: 10%, 15%, 20%, 25%, and 30%), various water content of cultural medium (40%, 45%, 50%, 55%, and 60%), and incubation duration in darkness (0, 3, 6, 9, and 12 days). In addition to the above test conditions, the standard solid medium with a wheat bran-to-chaff ratio of 8:2 and water content of 50% inoculated with 20% fermented optimal-liquid medium was incubated at 26 °C with a 12L/12D photoperiod for 10 days. The conidia yield was determined for different single factor conditions as described in above part.

### 2.5. Validity and Virulence of Stored B. bassiana PfBb Conidia Powder

This experiment was to detect the effect of conidia storage temperature on water content and conidial germination. The conidia powder produced by solid-state medium was maintained in vacuum-sealed bags and stored at one of the three test temperatures of −25, 4, and 20 °C, for 12 months. Three bags were sampled from each storage temperature monthly. To determine the conidia yield, 1 g BI powder was collected from each bag dissolved with 100 mL of 0.05% Tween-80 solution, and the conidia number was counted using the hemocytometer under the microscope. To assess the conidial germination rate, 1 g BI powder was dissolved in 0.05% Tween-80 solution, diluted to 1 × 10^7^ conidia/mL suspension, and then inoculated into liquid broth culture comprising 4% glucose and 1.5% yeast extract. The liquid broth culture was incubated at 26 °C for 24 h. The conidial germination rate was observed using a hemocytometer under the microscope, as mentioned in above part.

To determine the conidial water content after storage, a 1 g sample was collected from each bag and maintained in a glass bottle which was previously baked in an oven (PHG-9248A, Shanghai Jinghong Experimental Equipment Co., Ltd., Shanghai, China) at 120 °C for one hour. The conidia powder sample was oven dried for 2 h. The glass bottle containing conidia power was weighed before and after oven drying to determine the conidial water content.

All six larval instars of *P. flammans* were utilized to assess the virulence of *B. bassiana* PfBb conidia after storage at 4 °C for various durations (30, 60, 90, 120, and 360 days). There were four replicates for each storage duration. For each replicate, 15 larvae were individually sprayed with conidia powder for two seconds. Those conidia-infected larvae were placed in a Petri dish (20 cm diameter × 15 cm height) and fed with fresh *F. benjamina* leaves. The Petri dishes were maintained at 25 ± 1 °C and 75 ± 5% RH with a photoperiod of 13L/11D. They were checked twice daily (09:00 and 21:00) for seven consecutive days. Larvae were considered deceased if they did not respond when touched with a brush. The larval mortality rate was calculated. For each replicate, the dead larvae were transferred to a sterilized Petri dish. Larvae with mycelia growing on their integument were classified as cadavers. The number of larval cadavers was recorded, and the cadaver rate was calculated as (number of cadavers/total number of dead larvae) × 100%.

### 2.6. Statistical Analysis

Data were analyzed using SAS v.9.13 software (SAS Institute, Cary, NC, USA) with α = 0.05. The effect of storage duration on conidia virulence on different larval instars were analyzed using a generalized linear model (GLIMMIX procedure) followed by a binomial distribution and a logit function, with an LSD test for multiple comparisons. Data on the proportion of conidial water content and germination over varying storage duration at different temperatures were normally distributed (Shapiro–Wilk test, UNIVARIATE procedure) and analyzed using a general regression model (GLM procedure); the slopes of the regression models were compared by an analysis of covariance (ANCOVA, GLM procedure) with a ‘contrast’ statement for multiple comparisons. Other data were also normally distributed (Shapiro–Wilk test, UNIVARIATE procedure) and thus analyzed using ANOVA (GLM procedure) with an LSD test for multiple comparisons.

## 3. Results

### 3.1. Optimization of Liquid Fermentation for B. bassiana PfBb

Glucose as the carbon source had significantly higher mycelium dry weight (F_4,10_ = 76.08, *p* < 0.0001), and produced significantly more blastospores (F_4,10_ = 3.51, *p* = 0.0488) (Figure 1a). Yeast extract used as nitrogen sources in medium generated significantly higher mycelium dry weight (F_4,10_ = 309.92, *p* < 0.0001) and more blastospores (F_4,10_ = 152.28, *p* < 0.0001) (Figure 1b). The optimal concentrations of glucose and yeast extract were detected at 40 g/L (C:N 17:1) and 15 g/L (C:N 11.7:1), respectively (glucose: F_4,10_ = 125.26, *p* < 0.0001 for mycelium weight, and F_4,10_ = 4.09, *p* = 0.0323 for blastospore number; yeast extract: F_4,10_ = 8.73, *p* = 0.0027 for mycelium weight, and F_4,10_ = 22.40, *p* < 0.0001 for blastospore number) (Figure 1c,d).

Trace elements in the culture medium did not significantly promote *B. bassiana* PfBb growth and reproduction; rather, adding CoSO_4_, CaCl_2_, or CoCl_2_ significantly decreased mycelium weight (F_7,16_ = 35.53, *p* < 0.0001) and blastospore number (F_7,16_ = 8.55, *p* = 0.0002) (Figure 1e). In addition, increasing concentration of MgSO_4_, KCl, and MnSO_4_ had no significant effect on mycelium weight (F_7,16_ = 0.84, 0.64, and 0.80, respectively, *p* > 0.05), and increasing MgSO_4_ concentration had no significant effect on blastospore number (F_7,16_ = 0.78, *p* = 0.6130) (Figure 1f), while increased concentration of KCl and MnSO_4_ significantly decreased blastospore production concentration (F_7,16_ = 2.81 and 15.71, respectively, *p* < 0.05) (Figure 1g,h).

**Figure 2:**
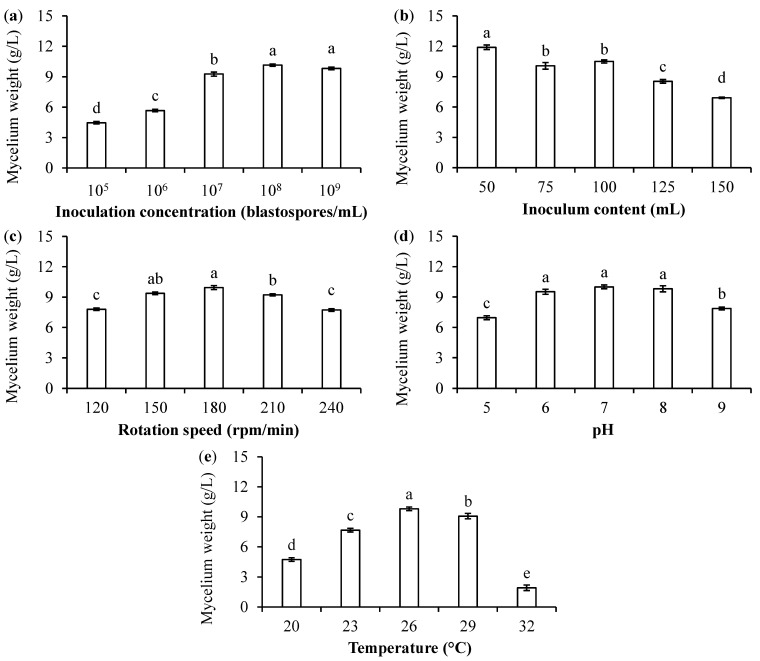
Mean (±SE) mycelium dry weight of *B. bassiana* PfBb growing in liquid culture medium affected by inoculation concentration of conidial suspension (**a**), inoculum content of liquid medium (**b**), rotation speed (**c**), pH value of liquid medium (**d**), and incubation temperature (**e**). Columns with the same letters are not significantly different (LSD test: *p* > 0.05).

The mycelium dry weight of *B. bassiana* PfBb was significantly affected by the inoculation concentration of the conidial suspension (F_4,10_ = 340.20, *p* < 0.0001), the inoculum content of the liquid medium (F_4,10_ = 85.73, *p* < 0.0001), the rotation speed (F_4,10_ = 48.83, *p* < 0.0001), the pH value of the liquid medium (F_4,10_ = 37.07, *p* < 0.0001), and the incubation temperature (F_4,10_ = 216.51, *p* < 0.0001), with the highest mycelium weight detected at an inoculation concentration of 1 × 10^8^ blastospore/mL (Figure 2a), inoculum content of 50 mL (Figure 2b), rotation speed of 180 rpm/min (Figure 2c), pH 7 (Figure 2d), and incubation temperature of 26 °C (Figure 2e).

### 3.2. Optimization of Solid Fermentation for B. bassiana PfBb

As shown in Table 1, conidia yield was significantly higher for the wheat bran medium than other medium types (F_4,10_ = 131.50, *p* < 0.0001), and the conidial germination rate was significantly higher for the wheat bran, corn flour, and rice mediums than for other medium types (F_4,10_ = 10.33, *p* = 0. 0014), suggesting that wheat bran medium was the best for *B. bassiana* PfBb growth.

**Figure 3:**
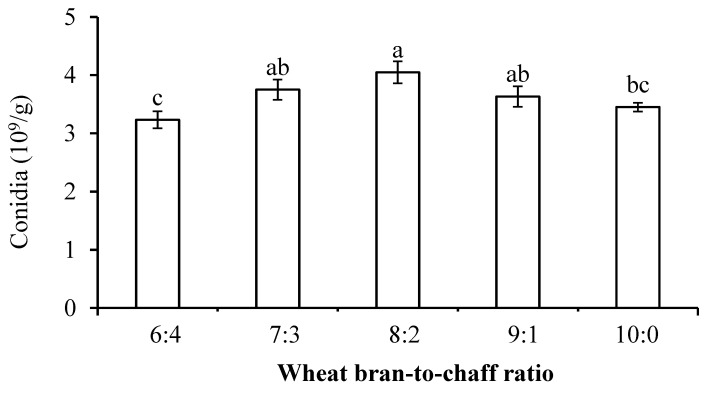
Mean (± SE) conidia production of *B. bassiana* PfBb affected by varying wheat bran-to-chaff ratios. Columns with the same letters are not significantly different (LSD test: *p* > 0.05).

Conidia yield was significantly higher for the chaff substrate than other substrate types (F_4,10_ = 20.13, *p* < 0.0001), and the conidial germination rate was significantly higher for the chaff and bagasse substrates than for other substrate types (F_4,10_ = 10.75, *p* = 0.0012) (Table 1), suggesting that chaff substrate was the best for *B. bassiana* PfBb growth. The optimal wheat bran-to-chaff ratio for *B. bassiana* PfBb growth was found to be 8:2 (F_4,10_ = 3.84, *p* = 0.0385) (Figure 3).

**Figure 4:**
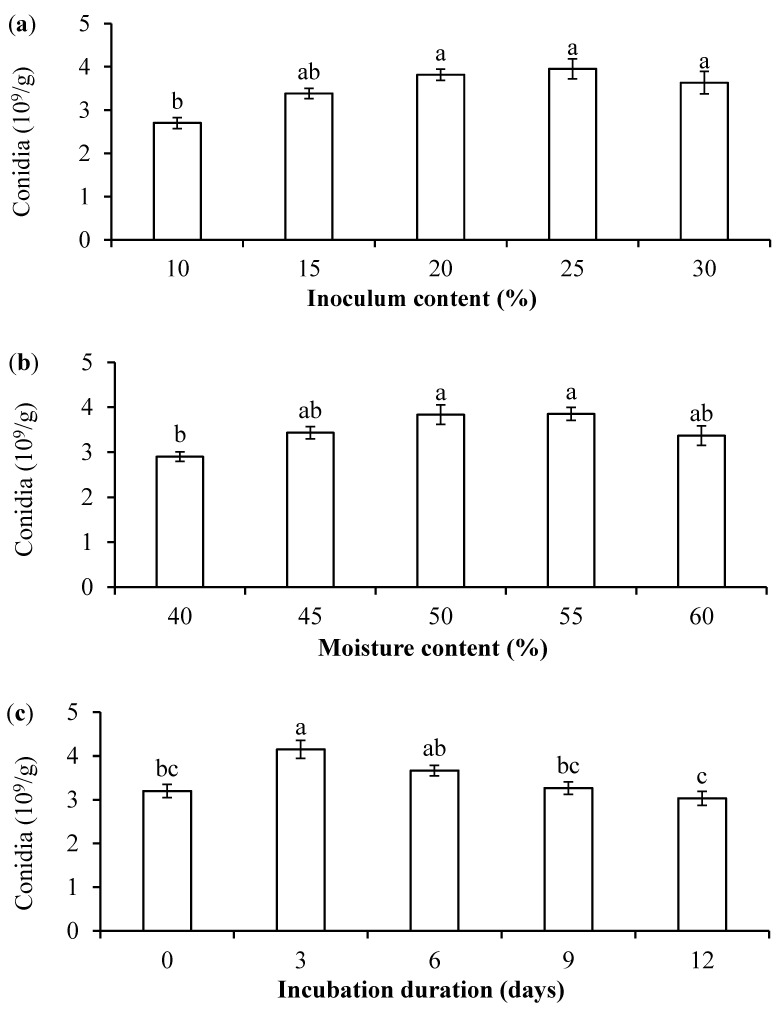
Effects of water content of solid medium (**a**), inoculum volume (**b**), and incubation duration in darkness (**c**) on conidia production of *B. bassiana* PfBb. Columns with the same letters are not significantly different (LSD test: *p* > 0.05).

The conidia production of *B. bassiana* PfBb was significantly affected by the inoculation volume of the medium (F_4,10_ = 7.23, *p* = 0.0053), the water content of the solid medium (F_4,10_ = 5.32, *p* = 0.0147), and the incubation duration in darkness (F_4,10_ = 8.11, *p* = 0.0035), with the highest conidia number detected at an inoculum content of 25% (Figure 4a), a 55% water content in medium (Figure 4b), and a dark incubation duration of 3 days (Figure 4c).

### 3.3. Validity and Virulence of Stored B. bassiana PfBb Conidia Powder

**Figure 5:**
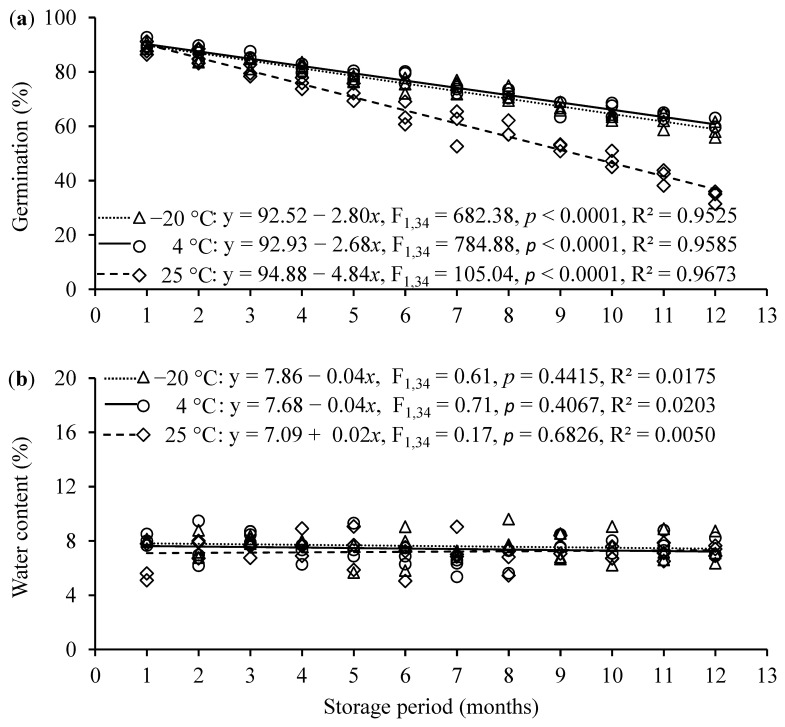
Effects of storage duration of *B. bassiana* PfBb conidia powder stored at different temperatures on the conidial germination rate (**a**), and conidia water content (**b**).

As shown in Figure 5, prolonging the storage duration of conidia power significantly decreased conidial germination rate at constant temperatures of −20, 4, and 20 °C (Figure 5a). The decreases in the conidial germination rate were significantly faster at 20 °C than at −20 and 4 °C (F_2,102_ = 100.41, *p* < 0.0001) (Figure 5a). However, the storage duration of conidia power had no significant impact on conidia water content, and there was no significant difference in the slopes of regression lines between the different storage temperatures (F_2,102_ = 0.50, *p* = 0.6086, Figure 5b).

Conidia stored at 4 °C for one year did not significantly decrease the virulence of *P. flammans* larvae on different instars, and there was no significant difference in larval mortality between instars for each storage duration, except that when conidia were stored for 360 days, the mortality of fifth and sixth instar larvae was significantly lower than that for first–fourth instars (Table 2). The cadaver rate of *P. flammans* larvae was not significantly different between different conidia storage durations for each larval instar, or between different larval instars for each storage duration (Table 3).

## 4. Discussion

The biphasic system, in which fungi are first grown under liquid-state conditions to produce metabolic active blastospores and then allowed to conidiate in solid-state conditions, is an efficient technology for mass production of *B. bassiana* for the biological control programs [14]. Usually, fungi can utilize a wide variety of nutrients sources present in the environment [30,41,68,69,70,71] and adapt to different environmental conditions [72,73,74]. This study optimized the nutrients, medium substrates, and fermentation environmental conditions for *B. bassiana* PfBb growth and conidiation, and developed measures to maintain storage validity and virulence and to prolong the shelf life of microbial pesticides, which are critical to the success of biological control programs using *B. bassiana* PfBb.

### 4.1. Optimization of Liquid Fermentation of B. bassiana PfBb

Carbon and nitrogen are the two main nutrients regulating fungal growth and production [26,28,33,34,75,76,77]. The amount of various carbon sources [68,78,79,80] and nitrogen sources [41,70,71,81,82], glucose, and yeast extract are highly favorable for *B. bassiana* growth in liquid fermentation [28,42,45,83,84,85]. Our results also indicate that liquid medium using glucose as the carbon source and yeast extract as the nitrogen source produced significantly higher mycelium mass and yielded more blastospores. Rangel et al. (2006) reported that glucose is the most utilizable carbon source for fungi, as it could be readily incorporated into the cell, and occupies the central position of the glycolytic pathway while other carbon sources are less preferred [78]. Furthermore, in many fungi, a process known as carbon and nitrogen catabolite repression may be involved in the efficient use of resources in the presence of nutrients, allowing the preferential utilization of glucose (or preferred sugars) and easily assimilated nitrogen sources (such as ammonium and glutamine) before other available carbon and nitrogen sources [76,86,87,88,89]. Therefore, our results suggest that glucose and yeast extracts were, respectively, the most favorable carbon and nitrogen sources in the liquid medium that facilitated the growth and production of *B. bassiana* PfBb.

Moreover, we showed that the lower and higher concentrations of glucose or yeast extract tended to reduce the mass growth and blastospore production of *B. bassiana* PfBb, with the optimal concentrations of glucose and yeast extract found to be 40% and 15%, respectively. It is reported that an increase in filamentous growth and blastospore production in *Metarhizium anisopliae* (Metchnikoff) Sorokin is associated with an increase in glucose concentration [90]. However, a very high glucose concentration may decrease the water activity of the medium, which will in turn reduce blastospore production [91]. Vega et al. (2003) and Safavi et al. (2007) reveal that the optimized carbon-to-nitrogen ratio is about 10:1 for the maximal fungal growth of *B. bassiana* [26,92]. In this study, the medium with C:N ratios of 17:1 (40 g glucose with 10 g yeast extract) and 11.7:1 (40 g glucose with 15 g yeast extract) facilitated mass growth and blastospore production, suggesting that the *B. bassiana* PfBb strain required less nitrogen to maximize the growth and reproduction compared to other strains. Nitrogen is the main input (cost) in mass production of entomopathogenic fungi [81]. Our results provide useful information to develop the cost-effective fermentation of *B. bassiana* PfBb in the liquid stage.

In addition to the macronutrients such as the carbon and nitrogen, some micronutrients, supplied at micromolar concentrations, including trace elements like calcium, copper, iron, manganese, and zinc, would be required for fungal cell growth [93,94,95]. It has been reported that the addition of MgSO_4_·7H_2_O, KCI, or MnSO_4_ promotes *B. bassiana* sporulation [30]. However, we find that the test micronutrients, including KCl, MgSO_4_, CaCl_2_, CuSO_4_, ZnSO_4_, MnSO_4_, or CoCl_2_, did not significantly promote *B. bassiana* PfBb growth and reproduction; on the contrary, CuSO_4_, CaCl_2_, and CoCl_2_ induced a negative impact on liquid fermentation of *B. bassiana* PfBb, and increasing the MgSO_4_ concentration had no significant effect on liquid fermentation or high concentrations of KCl and MnSO_4_, even significantly decreasing sporulation. We speculate that micronutrients affecting fungal growth and sporulation is strain-specific [96,97,98,99].

The mycelium weight increased significantly when the inoculation rate increased from 1 × 10^5^ to 1 × 10^8^ conidia/L, after which no significant increase in mycelium weight was observed, suggesting an optimal inoculation rate of 1 × 10^8^ conidia/L. However, mycelium weight decreased significantly when the inoculum content of liquid medium increased from 50 to 150 mL. Whether the inoculum content of 50 mL was the optimal one remains for further study. The decrease in mycelium weight with increasing inoculum content may be attributed to two reasons. First, the high inoculum content could lead to nutrient deficiency in the later fermentation stage, inhibiting mycelium growth [100,101,102]. Second, since the entomopathogenic fungi, including *B. bassiana*, are aerobic, the high inoculum content might reduce the amount of dissolved oxygen for liquid fermentation [103,104]. Agitation is thus necessary to improve the dissolution of oxygen in liquid fermentation. While a low rotation speed may result in a lack of oxygen in the culture medium [39,104], a high rotation speed can reduce enzyme production [98] and create shear forces among the suspended microbial cells in the liquid culture medium and cause cell damage by cell collision, resulting in a decrease in production [39,105,106]. We found an optimal rotation speed of 180 rpm/min for liquid fermentation of *B. bassiana* PfBb in this study.

The initial pH value is the critical factor affecting biomass accumulation [25]. Hallsworth and Magan (1996) report that a pH value ranging between 5 and 8 favors entomopathogenic fungal growth and conidial production; for example, in *B. bassiana*, *M. anisopliae*, and *Paecilomyces farinosus* [40]. The optimal pH value of 7 for maximal fungal growth found in the present study (Figure 1d) supported the study of Mishra and Malik [28]. In general, *B. bassiana* grows within a wide temperature range from 8 to 35 °C, with the optimal temperatures between 25 and 28 °C [23,107]. In this study, the optimal temperature for liquid fermentation of *B. bassiana* PfBb was 26 °C.

### 4.2. Optimization of Solid Fermentation of B. bassiana PfBb

For the mass production of entomopathogenic fungi for pest management programs, the selection of substrates and adoption of cultivation methods for solid fermentation may directly impact the cost and conidial yield. Many agricultural by-products and wastes can be employed as solid substrates in solid fermentation of *B. bassiana* PfBb, including rice bran, wheat bran, bagasse, coffee husks, and beer draff [27,83,108,109,110,111]. In this study, among the test solid substrates, wheat bran and chaff, respectively utilized as the nutritional and structural substrates, produced a significantly higher conidia yield and conidial germination rate. Previous research has indicated that wheat bran retains high levels of carbohydrates, starches, proteins, and minerals and is the most commonly utilized solid fermentation substrate for fungal production [110,112,113]. We reveal that the optimal wheat bran-to-chaff ratio for *B. bassiana* PfBb growth was at 8:2. Our results suggest that wheat bran supplied with sufficient nutrients and chaff with a wheat bran-to-chaff ratio of 8:2 performed the optimal physical structure that support sporulation.

The optimal inoculum content of fermented-liquid medium inoculated into the solid culture medium depends on the fungal strains and types of substrates. We show that when used wheat bran and chaff, the substrates with a ratio of 8:2, the conidia yield significantly increased with the increase of inoculum content from 10% to 20%, after which it did not increase at 25% inoculum with a decrease detected at 30% inoculum. The results agree with Camara et al. (2022) that 20–25% inoculum is optimal in solid fermentation of *B. bassiana* [45]. The decrease in conidia yield at 30% inoculum may be because that the moisture content of solid culture fermentation is usually maintained in the range of 12–70%, typically around 60% [29,45] and 50% water content with an inoculum of 20 mL conidial suspension generated the highest conidia yield; inoculating additional fermented-liquid medium (10% = 30–20%) would increase the moisture content of the solid substrate, reducing oxygen level and thus the conidia yield [114].

Additionally, lighting impacts sporulation in *B. bassiana* [43]. For instance, dark treatment during the initial stage of solid fermentation of *B. bassiana* fosters mycelium growth, whereas supplementary lighting during the conidia production stage enhances mycelium conidia production [115]. Zhang et al. (2014) reveal that the conidia production of *B. bassiana* in solid fermentation under continuous light is higher compared to that under intermittent lighting [72]. However, our results show that fermentation in darkness for three days produced significantly higher conidia yield, and similar conidia yield was recorded when solid medium was fermented for 12–16 days under a 12L/12D photoperiod (unpublished data). Therefore, fermentation in darkness will shorten the duration and significantly reduce the costs of mass production.

In addition to the successful mass production of fungal biopesticides, maintaining the validity and virulence of conidia for 12–18 months before utilization is critical to the implementation of biological control programs [38,56,116,117]. Previous studies have shown that low temperatures are more favorable for the long-term preservation of *B. bassiana* conidia powder [38], while high temperatures may lead to the reduction in conidia viability and virulence of *B. bassiana* [118,119]. In the present study, when *B. bassiana* PfBb conidia were stored in vacuum-sealed bags at −20, 4, or 25 °C, the conidial germination rate significantly decreased over the 12-month storage duration; however, the decrease in germination rate was significantly lower at −20 and 4 °C than at 25 °C. Therefore, storing *B. bassiana* PfBb conidia at a temperature slightly above freezing is feasible. The higher germination rate at −20 and 4 °C may be partially attributed to the constant lower water content of conidia over the storage duration, as a lower water content (<12%) is required to maintain the higher survival rate, stability, and virulence of *B. bassiana* PfBb conidia [120].

### 4.3. Validity and Virulence of Stored B. bassiana PfBb Conidia Powder

We further reveal that conidia were stored at 4 °C for one year, and the mortality rate *P. flammans* larvae and the cadaver rates of larvae were not significantly different between conidia storage durations for each larval instar, or between the six larval instars for each storage duration, except that conidia stored for one year induced a significantly lower mortality of fifth and sixth instar larvae than of first–fourth instars. Our previous study demonstrates that the toxicity of *B. bassiana* PfBb to the *S. frugiperda* larvae and the cadaver rate of larvae significantly decrease with the increase in instar stage [65]. Our results have three implications: (1) storage at 4 °C could maintain the virulence of *B. bassiana* PfBb conidia for a long period of time, (2) the high cadaver rate of dead larvae will enable the spread and insistence of *B. bassiana* PfBb in fields, and (3) *B. bassiana* PfBb has a high potential for the biological control of rate *P. flammans*. Further experiments will test the toxicity of *B. bassiana* PfBb to other target and non-target pest species in order to determine if this biopesticide could be applied for large-scale pest management.

The cause of reduced mortality of fifth and sixth instar larvae exposed to one-year-old *B. bassiana* PfBb conidia may be that a prolonged storage period might reduce the germination capacity or virulence of conidia due to metabolic stress [17,20]. Furthermore, late-instar larvae of moths usually have thicker cuticles [121,122,123] and often develop more robust immune systems (e.g., melanization, hemocyte activity) [122,124], which can decrease the possibility of fungal penetration and colonization, respectively. In addition, older instar larvae may exhibit stronger grooming behaviors or avoid contaminated surfaces, reducing contact with conidia [125,126]. Therefore, a higher dose of viable conidia is required to achieve a higher mortality of older larvae; this remains for further investigations.

## 5. Conclusions

We find that in liquid fermentation, glucose, and yeast extract with a C:N ratio of 17:1 are the best carbon and nitrogen sources for *B. bassiana* PfBb growth and blastospore production, and the inoculation concentration of 1 × 10^8^/mL and inoculum content of 50 mL conidial suspension, 180 rpm/min rotation speed, pH of 7, and temperature of 26 °C create the optimal medium conditions for liquid fermentation. In solid fermentation, wheat bran and chaff with a ratio of 8:2 optimize *B. bassiana* PfBb sporulation and conidial germination, and the inoculum content of 25%, 55% water content in medium, and 3-day fermentation in darkness produce the highest conidia yield. The resulting biopesticides stored at 4 °C for one year will maintain the validity and virulence of stored *B. bassiana* PfBb conidia, which are toxic to all instar larvae of *P. flammans*. Our results provide essential information to improve the production of commercial biopesticides based on *B. bassiana* PfBb.

## Figures and Tables

**Table 1:** Effect of different solid substrates on *B. bassiana* PfBb conidia yield and germination.

Substrate	Conidia Yield (10^9^/g)	Germination (%)
Nutritional substrate		
Wheat bran	4.45 ± 0.08 ^a^	93.01 ± 0.82 ^a^
Corn flour	3.15 ± 0.13 ^b^	93.52 ± 0.24 ^a^
Rice	2.37 ± 0.15 ^c^	93.73 ± 0.47 ^a^
Soybean meal	1.57 ± 0.07 ^d^	90.04 ± 1.06 ^b^
Beer draff	1.18 ± 0.13 ^e^	87.94 ± 0.96 ^b^
Structural substrate		
Chaff	1.68 ± 0.07 ^a^	94.55 ± 1.20 ^a^
Bagasse	1.20 ± 0.09 ^b^	93.01 ± 0.52 ^a^
Coir	0.75 ± 0.13 ^c^	89.03 ± 1.69 ^b^
Peanut shell	0.67 ± 0.09 ^c^	86.20 ± 0.98 ^b^
Tobacco stem	0.73 ± 0.09 ^c^	86.48 ± 1.08 ^b^

For the nutritional or structural substrates, means (± SE) with the same letters in a column are not significantly different (LSD test: *p* > 0.05).

**Table 2:** Effect of storage duration (days) of *B. bassiana* PfBb conidia powder on mortality (%) of *P. flammans* larvae of different instars.

Storage				Larval Instar			F_5,18_	*p*
Duration (d)	1st	2nd	3rd	4th	5th	6th		
30	100	100	100	100	95.00 ± 3.19	85.00 ± 1.67	0.61	0.6948
60	100	100	100	100	95.00 ± 3.19	81.67 ± 3.19	0.91	0.4950
90	100	100	100	91.67 ± 3.19	93.33 ± 2.72	83.33 ± 3.33	0.69	0.6350
120	100	100	100	100	93.33 ± 2.72	80.00 ± 6.08	0.84	0.5362
150	100	100	100	98.33 ± 1.67	91.67 ± 3.18	83.33 ± 1.92	1.24	0.3303
180	100	100	100	91.75 ± 5.00	85.00 ± 4.19	71.67 ± 4.19	1.61	0.2076
360	100 ^a^	100 ^a^	98.33 ± 1.67 ^a^	96.67 ± 3.33 ^a^	78.33 ± 4.19 ^b^	63.33 ± 5.77 ^b^	4.64	0.0068 *
F_6,21_	0	0	0	0.59	2.40	2.14		
P	1	1	1	0.7318	0.0634	0.0918		

* Means (±SE) with the same letters in the row for storage duration of 360 days were not significantly different (LSD test: *p* > 0.05).

**Table 3:** Effect of storage duration (days) of *B. bassiana* PfBb conidia powder on larval cadaver (%) of *P. flammans* larvae of different instars.

Storage				Larval Instar			F_5,18_	*p*
Duration (d)	1st	2nd	3rd	4th	5th	6th		
30	95.00 ± 1.67	95.00 ± 3.19	98.33 ± 1.67	91.67 ± 3.19	92.96 ± 2.73	90.06 ± 3.86	0.73	0.6116
60	93.33 ± 2.76	93.33 ± 3.85	95.00 ± 3.19	86.67 ± 2.72	86.03 ± 2.76	93.88 ± 3.76	1.09	0.3995
90	91.67 ± 3.19	90.00 ± 4.30	91.67 ± 4.19	94.74 ± 3.25	89.78 ± 6.12	90.18 ± 3.17	0.68	0.6424
120	91.67 ± 4.19	90.00 ± 4.30	90.00 ± 4.30	88.33 ± 3.19	87.21 ± 3.85	90.31 ± 3.51	0.13	0.9833
150	96.67 ± 1.92	93.33 ± 2.72	95.00 ± 3.19	89.76 ± 2.07	92.82 ± 2.73	91.83 ± 3.41	0.51	0.7676
180	95.00 ± 1.67	95.00 ± 1.67	91.67 ± 3.19	95.26 ± 5.87	92.10 ± 0.42	93.37 ± 2.22	0.27	0.9239
360	95.00 ± 3.19	91.67 ± 3.19	95.00 ± 3.19	91.92 ± 5.92	91.57 ± 3.19	92.95 ± 4.40	0.62	0.6878
F_6,21_	0.37	0.38	0.0.73	0.46	0.72	0.16		
P	0.8902	0.8806	0.6316	0.8299	0.6360	0.9835		

## Data Availability

The original contributions presented in this study are included in the article. Further inquiries can be directed to the corresponding author.
